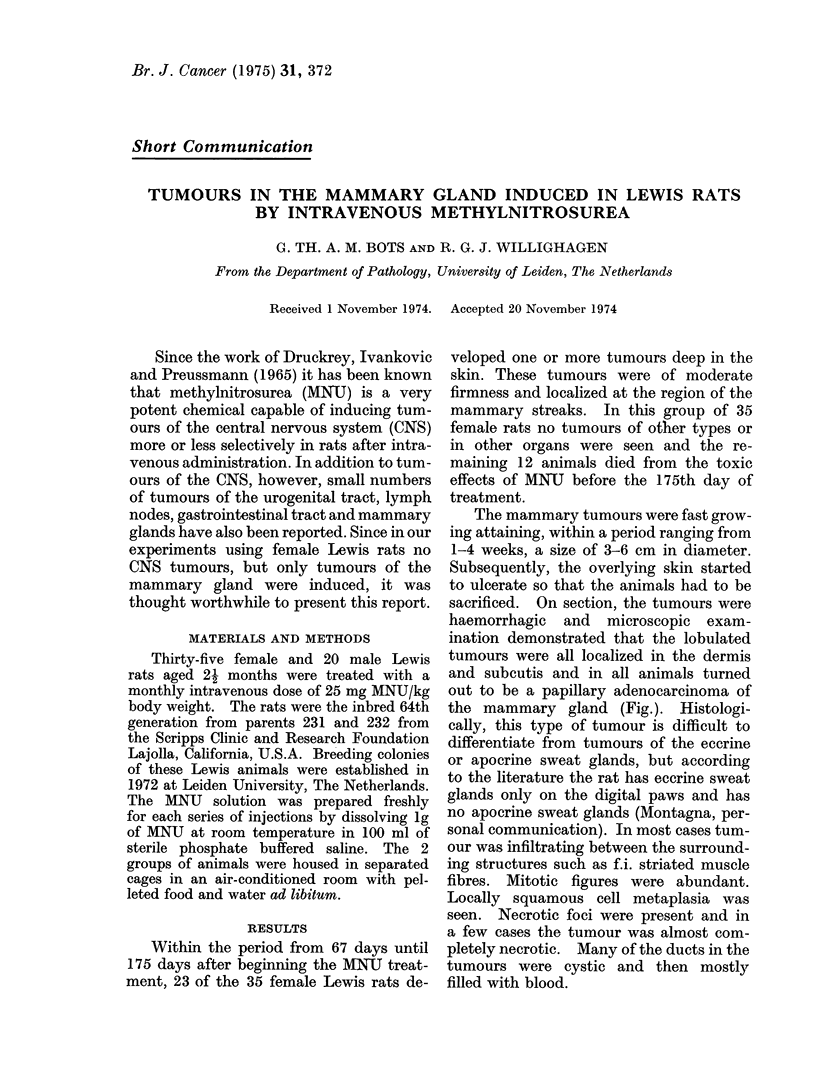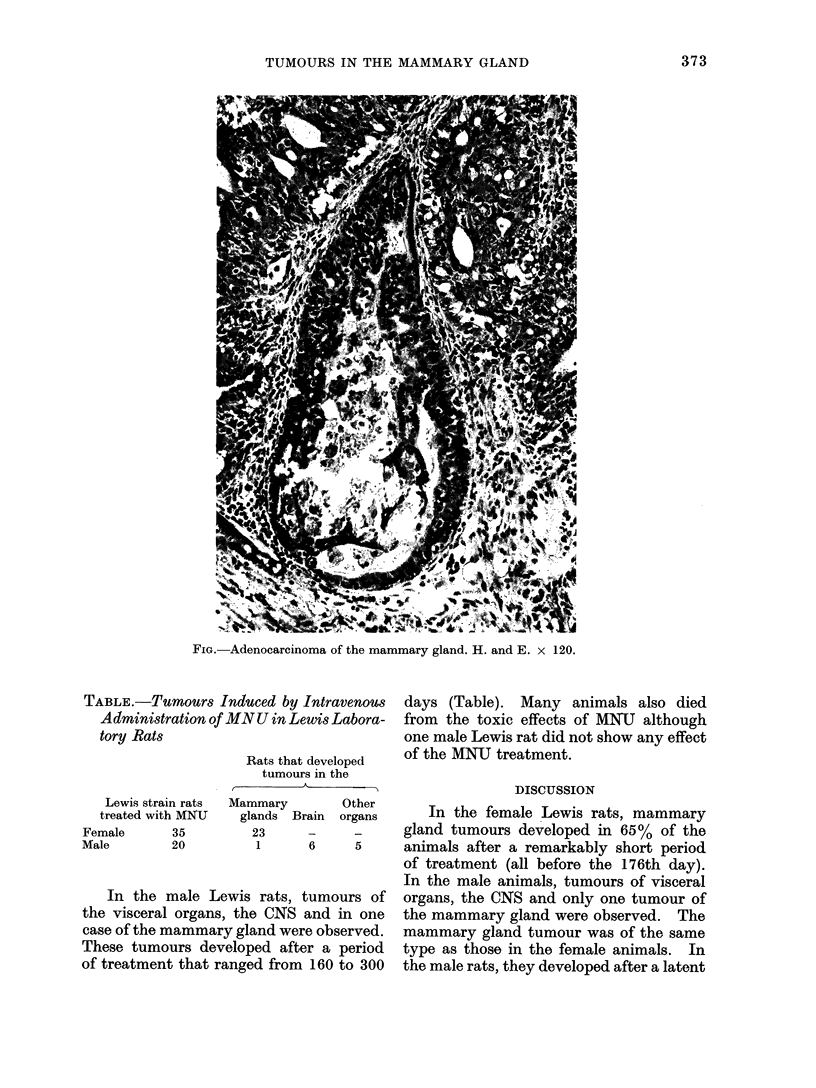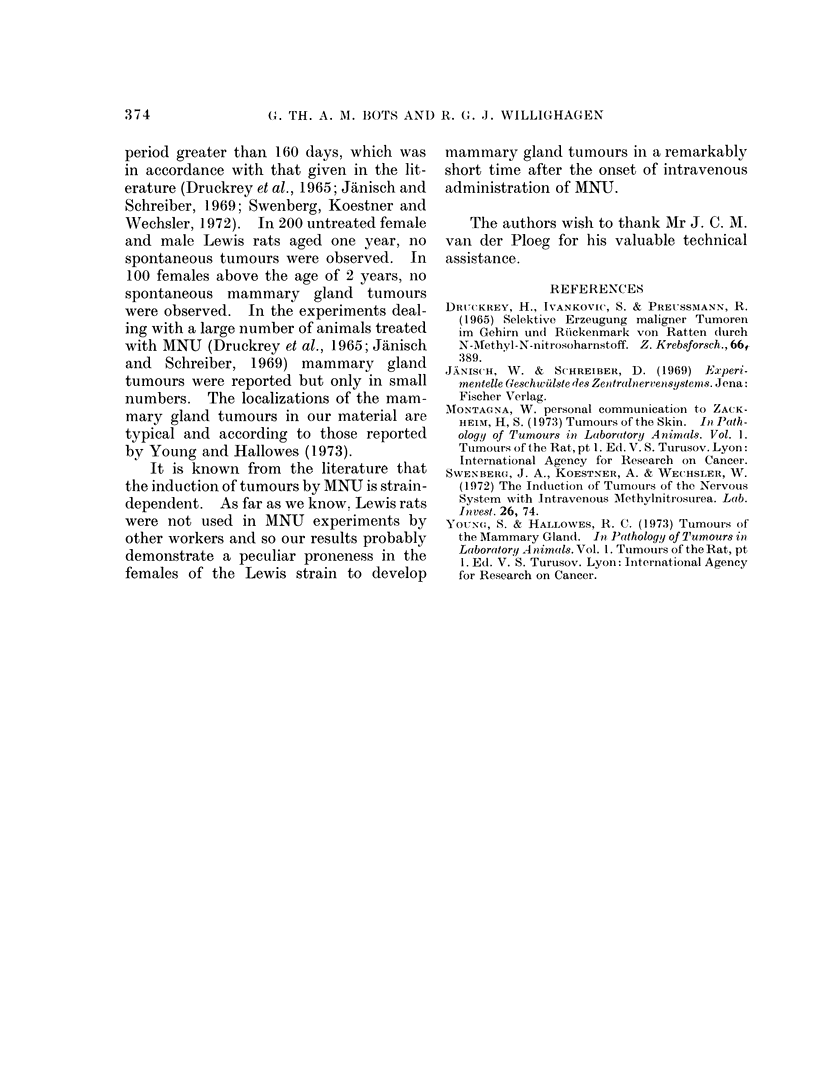# Tumours in the mammary gland induced in Lewis rats by intravenous methylnitrosurea.

**DOI:** 10.1038/bjc.1975.74

**Published:** 1975-03

**Authors:** G. T. Bots, R. G. Willighagen

## Abstract

**Images:**


*Br. J. Cancer* (1975) **31**, 372

***Short Communication***

### TUMOURS IN THE MAMMARY GLAND INDUCED IN LEWIS RATS BY INTRAVENOUS METHYLNITROSUREA

G. TH. A. M. BOTS AND R. G. J. WILLIGHAGEN

*From the Department of Pathology, University of Leiden, The Netherlands*

Received 1 November 1974. Accepted 20 November 1974

Since the work of Druckrey, Ivankovic and Preussmann (1965) it has been known that methylnitrosurea (MNU) is a very potent chemical capable of inducing tumours of the central nervous system (CNS) more or less selectively in rats after intravenous administration. In addition to tumours of the CNS, however, small numbers of tumours of the urogenital tract, lymph nodes, gastrointestinal tract and mammary glands have also been reported. Since in our experiments using female Lewis rats no CNS tumours, but only tumours of the mammary gland were induced, it was thought worthwhile to present this report.

### MATERIALS AND METHODS

Thirty-five female and 20 male Lewis rats aged $2\frac{1}{2}$ months were treated with a monthly intravenous dose of 25 mg MNU/kg body weight. The rats were the inbred 64th generation from parents 231 and 232 from the Scripps Clinic and Research Foundation Lajolla, California, U.S.A. Breeding colonies of these Lewis animals were established in 1972 at Leiden University, The Netherlands. The MNU solution was prepared freshly for each series of injections by dissolving 1g of MNU at room temperature in 100 ml of sterile phosphate buffered saline. The 2 groups of animals were housed in separated cages in an air-conditioned room with pelleted food and water *ad libitum*.

### RESULTS

Within the period from 67 days until 175 days after beginning the MNU treatment, 23 of the 35 female Lewis rats developed one or more tumours deep in the skin. These tumours were of moderate firmness and localized at the region of the mammary streaks. In this group of 35 female rats no tumours of other types or in other organs were seen and the remaining 12 animals died from the toxic effects of MNU before the 175th day of treatment.

The mammary tumours were fast growing attaining, within a period ranging from 1–4 weeks, a size of 3–6 cm in diameter. Subsequently, the overlying skin started to ulcerate so that the animals had to be sacrificed. On section, the tumours were haemorrhagic and microscopic examination demonstrated that the lobulated tumours were all localized in the dermis and subcutis and in all animals turned out to be a papillary adenocarcinoma of the mammary gland (Fig.). Histologically, this type of tumour is difficult to differentiate from tumours of the eccrine or apocrine sweat glands, but according to the literature the rat has eccrine sweat glands only on the digital paws and has no apocrine sweat glands (Montagna, personal communication). In most cases tumour was infiltrating between the surrounding structures such as f.i. striated muscle fibres. Mitotic figures were abundant. Locally squamous cell metaplasia was seen. Necrotic foci were present and in a few cases the tumour was almost completely necrotic. Many of the ducts in the tumours were cystic and then mostly filled with blood.

FIG.—Adenocarcinoma of the mammary gland. H. and E. × 120.

TABLE.—*Tumours Induced by Intravenous Administration of MNU in Lewis Laboratory Rats*

| Lewis strain rats treated with MNU | | Rats that developed tumours in the Mammary glands | Brain | Other organs |
|---|---|---|---|---|
| Female | 35 | 23 | – | – |
| Male | 20 | 1 | 6 | 5 |

In the male Lewis rats, tumours of the visceral organs, the CNS and in one case of the mammary gland were observed. These tumours developed after a period of treatment that ranged from 160 to 300 days (Table). Many animals also died from the toxic effects of MNU although one male Lewis rat did not show any effect of the MNU treatment.

### DISCUSSION

In the female Lewis rats, mammary gland tumours developed in 65% of the animals after a remarkably short period of treatment (all before the 176th day). In the male animals, tumours of visceral organs, the CNS and only one tumour of the mammary gland were observed. The mammary gland tumour was of the same type as those in the female animals. In the male rats, they developed after a latent period greater than 160 days, which was in accordance with that given in the literature (Druckrey *et al.*, 1965; Jänisch and Schreiber, 1969; Swenberg, Koestner and Wechsler, 1972). In 200 untreated female and male Lewis rats aged one year, no spontaneous tumours were observed. In 100 females above the age of 2 years, no spontaneous mammary gland tumours were observed. In the experiments dealing with a large number of animals treated with MNU (Druckrey *et al.*, 1965; Jänisch and Schreiber, 1969) mammary gland tumours were reported but only in small numbers. The localizations of the mammary gland tumours in our material are typical and according to those reported by Young and Hallowes (1973).

It is known from the literature that the induction of tumours by MNU is strain-dependent. As far as we know, Lewis rats were not used in MNU experiments by other workers and so our results probably demonstrate a peculiar proneness in the females of the Lewis strain to develop mammary gland tumours in a remarkably short time after the onset of intravenous administration of MNU.

The authors wish to thank Mr J. C. M. van der Ploeg for his valuable technical assistance.

### REFERENCES

DRUCKREY, H., IVANKOVIC, S. & PREUSSMANN, R. (1965) Selektive Erzeugung maligner Tumoren im Gehirn und Rückenmark von Ratten durch N-Methyl-N-nitrosoharnstoff. *Z. Krebsforsch.*, **66**, 389.

JÄNISCH, W. & SCHREIBER, D. (1969) *Experimentelle Geschwülste des Zentralnervensystems.* Jena: Fischer Verlag.

MONTAGNA, W. personal communication to ZACKHEIM, H, S. (1973) Tumours of the Skin. *In Pathology of Tumours in Laboratory Animals. Vol.* 1. Tumours of the Rat, pt 1. Ed. V. S. Turusov. Lyon: International Agency for Research on Cancer.

SWENBERG, J. A., KOESTNER, A. & WECHSLER, W. (1972) The Induction of Tumours of the Nervous System with Intravenous Methylnitrosurea. *Lab. Invest.* **26**, 74.

YOUNG, S. & HALLOWES, R. C. (1973) Tumours of the Mammary Gland. *In Pathology of Tumours in Laboratory Animals.* Vol. 1. Tumours of the Rat, pt 1. Ed. V. S. Turusov. Lyon: International Agency for Research on Cancer.